# 25. Immunogenicity and Reactogenicity of COVID-19 mRNA Vaccines in Allogeneic Stem Cell Transplant Recipients

**DOI:** 10.1093/ofid/ofab466.025

**Published:** 2021-12-04

**Authors:** Bruce P Bausk, Amy C Sherman, Michaël Desjardins, Natalie E Izaguirre, Chi-An Cheng, Megan Powell, Yasmeen Senussi, Tal Gilboa, Jonathan H Krauss, Bonnie Dirr, Elyssa Power, Amy Joyce, Lisa Stewart, Omolola Ometoruwa, Lewis A Novack, Bethany Evans, Tenaizus Woods, Alexandra Tong, David Walt, Robert Soiffer, Vincent T Ho, Nicolas C Issa, Lindsey R Baden

**Affiliations:** 1 Brigham and Women’s Hospital, Boston, MA; 2 Harvard Medical School/Brigham and Women’s Hospital, Boston, Massachusetts; 3 Brigham and Women’s Hospital, Boston, Massachusetts; 4 BWH Division of Infectious Diseases, Boston, Massachusetts; 5 Brigham and Womens' hospital, Brookline, Massachusetts; 6 Brigham & Women’s Hospital, Boston, Massachusetts; 7 Dana Farber Cancer Institute, Boston, Massachusetts; 8 Brigham and Womens Hospital, Boston, Massachusetts; 9 Harvard Medical School/Brigham and Women’s Hospital/Wyss Institute, Boston, Massachusetts; 10 DFCI, boston, Massachusetts; 11 Dana-Farber Cancer Institute, Boston, Massachusetts

## Abstract

**Background:**

Allogeneic stem cell transplant (SCT) recipients are at an increased risk of poor outcomes from COVID-19. While the mRNA-1273 (Moderna) and BNT162b2 (Pfizer) COVID-19 mRNA vaccines are highly immunogenic in the general population, the immune response in SCT recipients is poorly understood. We characterized the immunogenicity and reactogenicity of COVID-19 mRNA vaccines in a cohort of SCT patients.

**Methods:**

We performed a prospective cohort study of 16 allogeneic SCT patients and 23 healthy controls. Blood samples for both cohorts were collected prior to first vaccination (baseline), at the time of second vaccination, and approximately 28 days post-second vaccination. Anti-Spike (S), anti-S1, anti-receptor binding domain (RBD), and anti-Nucleocapsid (N) IgG levels were measured quantitatively from plasma using a multiplexed single molecule array (Simoa) immunoassay. Reactogenicity was captured for the SCT cohort via a self-reported post-vaccination diary for 7 days after each dose.

**Results:**

Demographics and SCT recipients’ characteristics are shown in Table 1. In the SCT cohort, we observed a significantly lower anti-S (p< 0.0001), S1 (p< 0.0001), and RBD (p< 0.0001) IgG responses as compared to healthy controls, both at the time of dose 2 and 28 days post-vaccine series (Fig 1). Overall, 62.5% of SCT recipients were responders after vaccine series completion, as compared to 100% of healthy controls (Fig 2). While no patients had a reported history of COVID-19 diagnosis, 2 patients in the SCT cohort had elevated anti-S IgG levels and 1 showed elevated anti-N at baseline.

10/16 participants in the SCT cohort completed at least one post-vaccination diary. Local and systemic reactions were reported by 67% and 22% of participants, respectively, after dose 1, and 63% and 50% after dose 2 (Figure 3). All reported events were mild.

Table 1: Demographics

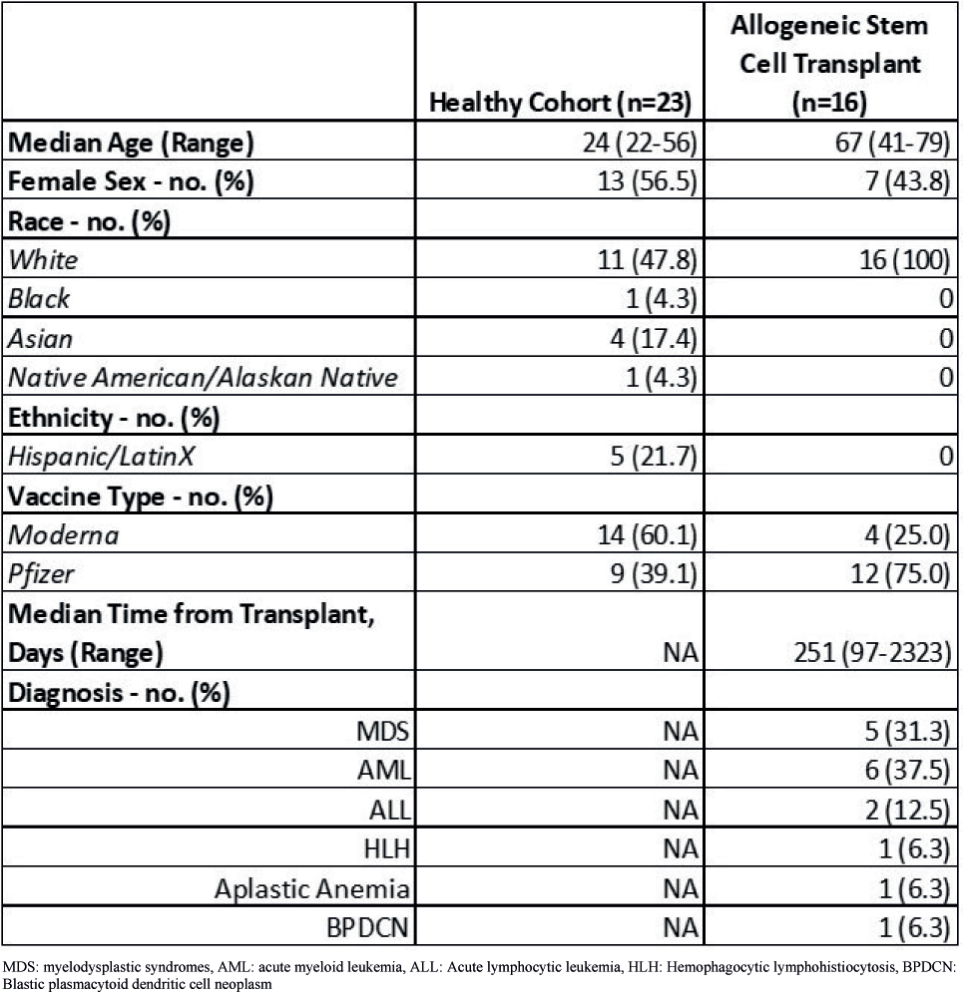

Figure 1: Plasma IgG Titers

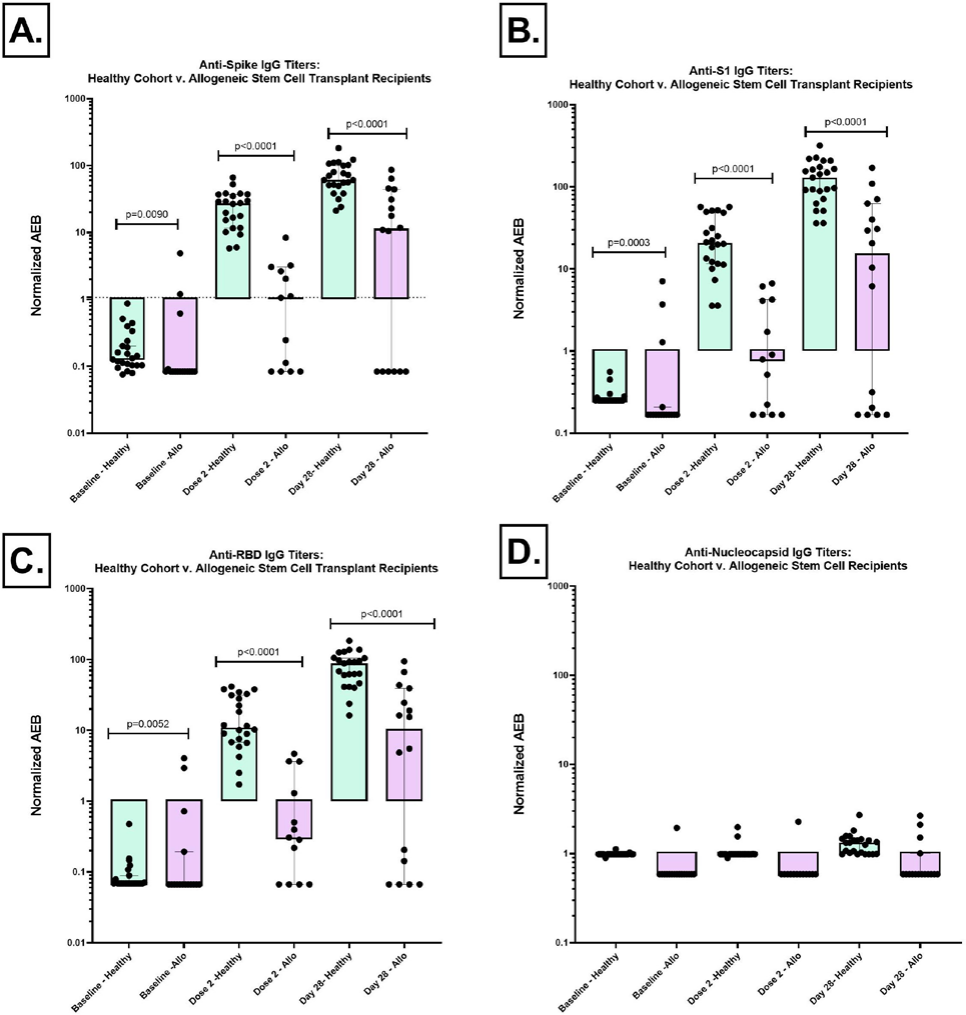

Anti-Spike (A), anti-S1 (B), anti-RBD (C), and anti-nucleocapsid (D) IgG titers were measured at baseline, time of second dose, and approximately 28 days after second vaccination. IgG levels were measured quantitatively using multiplexed single molecule array (Simoa) immunoassays, and are reported as Normalized Average Enzymes per Bead (AEB). Allogeneic stem cell transplant recipients (mauve) showed significantly lower anti-S, S1, and RBD IgG responses as compared to healthy controls (mint). Low titers of anti-N IgG demonstrates no history of COVID-19 natural infection during the course of the study.

Figure 3. Solicited Local and Systemic Adverse Events

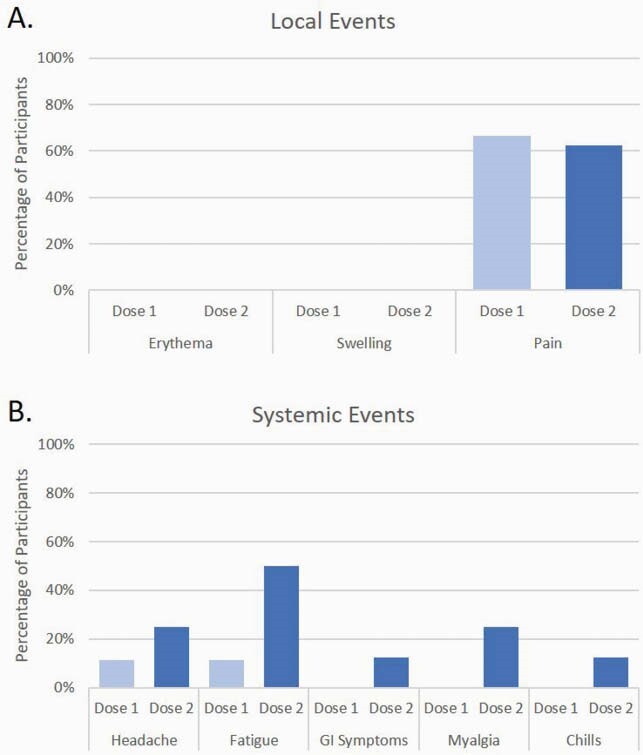

10 allogeneic stem cell transplant recipients completed at least one diary for 7 days after vaccination. Reactions after dose 1 are shown in light blue, and reactions after dose 2 are shown in dark blue. Local reactions (A) were reported by 67% (6/9) of participants after dose 1, and 63% (5/8) after dose 2. Systemic reactions (B) were reported by 22% (2/9) of participants after dose 1, and 50% (4/8) after dose 2. All reported events were mild (Grade 1).

**Conclusion:**

Among SCT recipients, mRNA COVID-19 vaccines were well-tolerated but less immunogenic than in healthy controls. Further study is warranted to better understand heterogeneous characteristics that may affect the immune response in order to optimize COVID-19 vaccination strategies for SCT recipients.

Figure 2: Response Rate to COVID-19 Vaccination

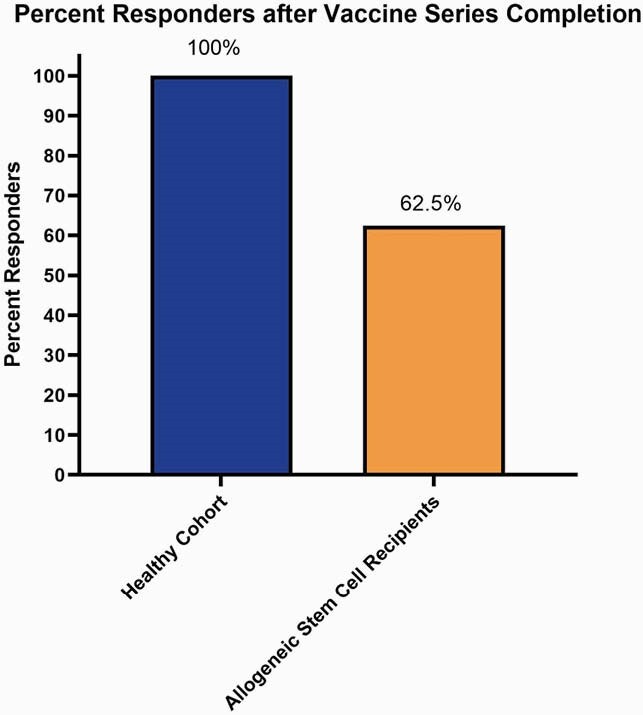

An internally validated threshold for responders was established using pre-pandemic sera from healthy adults. A positive antibody response was was defined as individuals with anti-Spike IgG levels above the 1.07 Normalized AEB threshold.

**Disclosures:**

**Amy Joyce, NP**, **Kadmon** (Advisor or Review Panel member) **Lewis A. Novack, MS**, **Lumicell Inc.** (Scientific Research Study Investigator, Research Grant or Support)**Precision Healing, Inc.** (Scientific Research Study Investigator, Research Grant or Support) **David Walt, PhD**, **Quanterix Corporation** (Board Member, Shareholder) **Robert Soiffer, MD**, **alexion** (Consultant)**gilead** (Advisor or Review Panel member)**jazz** (Advisor or Review Panel member)**juno/bms** (Advisor or Review Panel member)**kiadis** (Board Member)**precision bioscience** (Consultant)**Rheos** (Consultant)**takeda** (Consultant) **Nicolas C. Issa, MD**, **AiCuris** (Scientific Research Study Investigator)**Astellas** (Scientific Research Study Investigator)**GSK** (Scientific Research Study Investigator)**Merck** (Scientific Research Study Investigator)

